# Supplementary material for: The Penta-EF-Hand ALG-2 Protein Interacts with the Cytosolic Domain of the SOCE Regulator SARAF and Interferes with Ubiquitination
Source: Int J Mol Sci. 2020 Aug 31;21(17):6315. doi: 10.3390/ijms21176315 (PMC7504102; doi:10.3390/ijms21176315)
Supplement: Supplementary file 1 [file ijms-21-06315-s001.zip › SupplFigs.pdf]

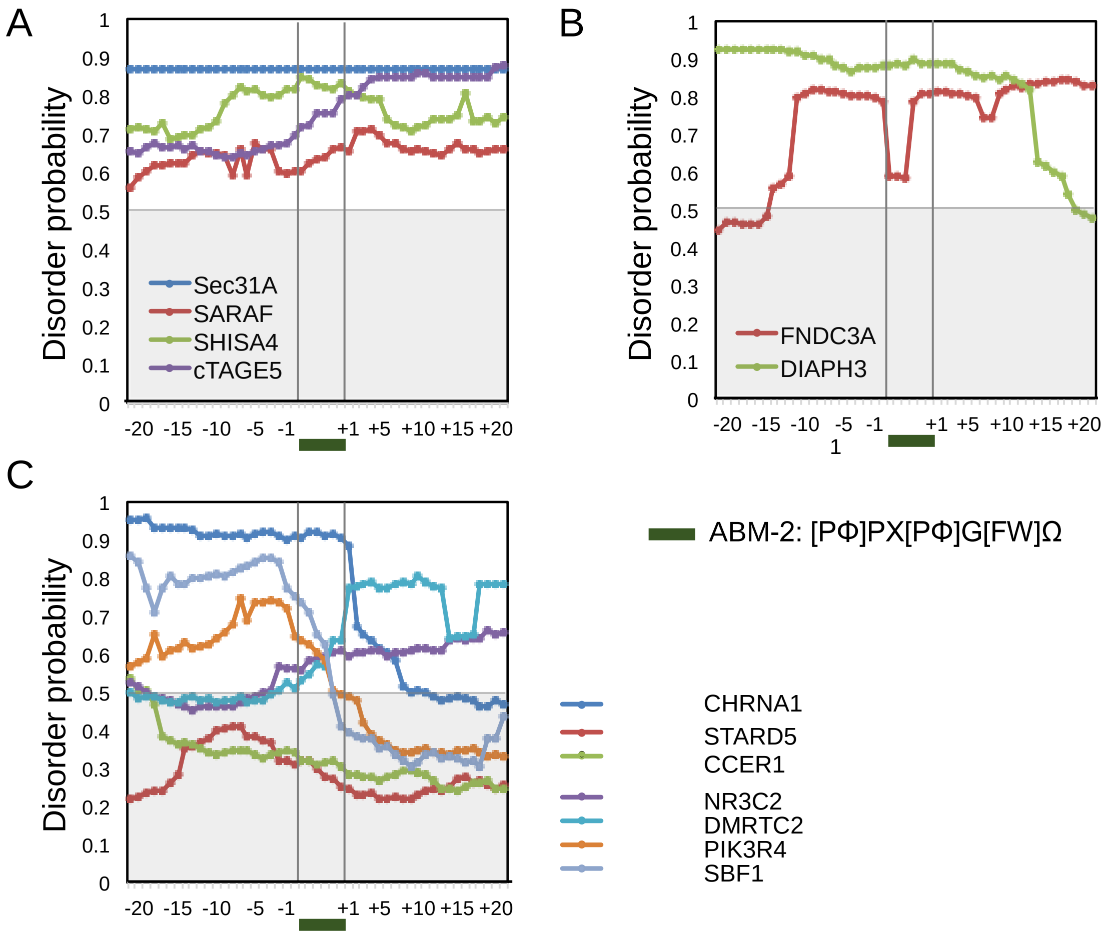

Supplementary Figure S1. Predicted intrinsically disordered regions. Disorder probability of proteins containing ALG-2-binding motif 2 (ABM-2) was predicted from amino acid sequences by the GeneSilico MetaDisorder service at the following URL site: http://iimcb.genesilico.pl/metadisorder/. A disorder probability above 0.5 is regarded as intrinsically unfolded. Results of the metadisorder program of MD2 are shown. Proteins displayed in A (interaction, positive), B (interaction, positive or negative), and C (interaction, negative) correspond to the groups shown in Supplementary Table S1.

Reference:

Kozlowski LP, Bujnicki JM. MetaDisorder: a meta-server for the prediction of intrinsic disorder in proteins. BMC Bioinformatics. 2012, 13(1):111. doi: 10.1186/1471-2105-13-111

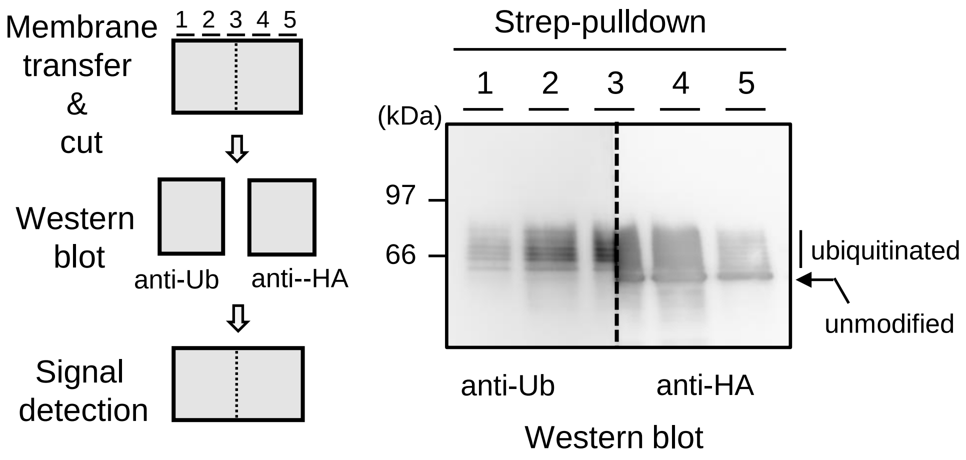

Supplementary Figure S2. Western blot signals detected with anti-ubiquitin antibody for slower migrating bands. After Strep-pulldown products used in Figure 4 had been resolved by SDS-PAGE using different amounts (lanes 1 and 5, one fourth of the amounts applied to lanes 2, 3, and 4), the blotted membrane was cut into halves in the middle of lane 3 and immuno-reacted with specific antibodies against ubiquitin (Ub) and HA, respectively. For detection of chemiluminescent signals, membranes were placed side by side in contact with each other. Left panel, flow chart of manipulation. Right panel, WB data. Arrow, unmodified proteins (molecular mass calculated from the amino acid sequence, 55.2 kDa).

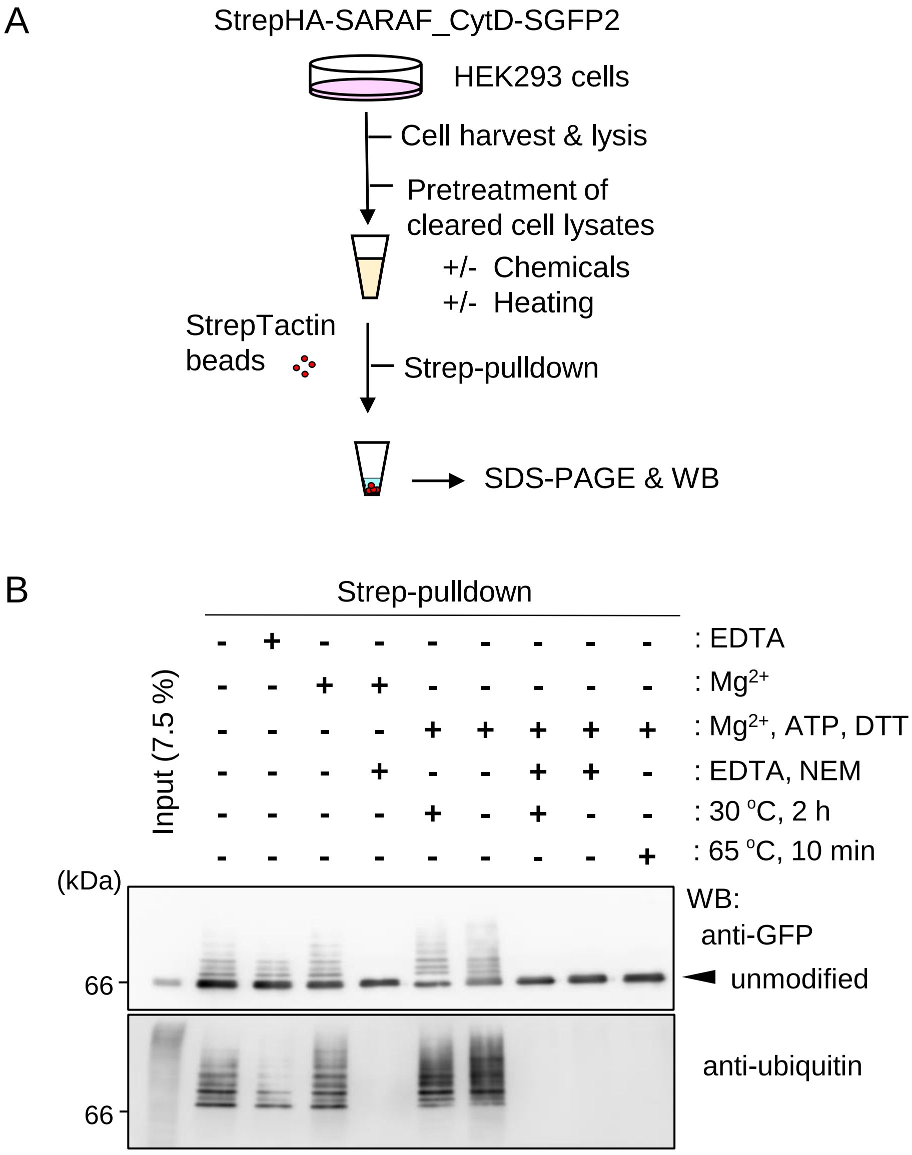

Supplementary Figure S3. (**A**) Flowchart of experiments. (**B**) HEK293 cells expressing StrepHA-SARAF_CytD-SGFP2 were lysed with lysis buffer HK (20 mM HEPES-NaOH, pH 7.4, 142.5 mM KCl) containing 0.2% Nonidet P-40 and protease inhibitors and the cleared lysates were subjected to Strep-pulldown either after heat treatment at 30 °C for 2 h or at 65 °C for 10 min or without heat treatment in the presence of different combinations of supplemental chemicals (2 mM EDTA, 1.5 mM $MgCl_2$, 3 mM ATP, 0.2 mM DTT, 10 mM NEM). Pulldown products were analyzed by WB with anti-GFP and anti-ubiquitin as indicated.

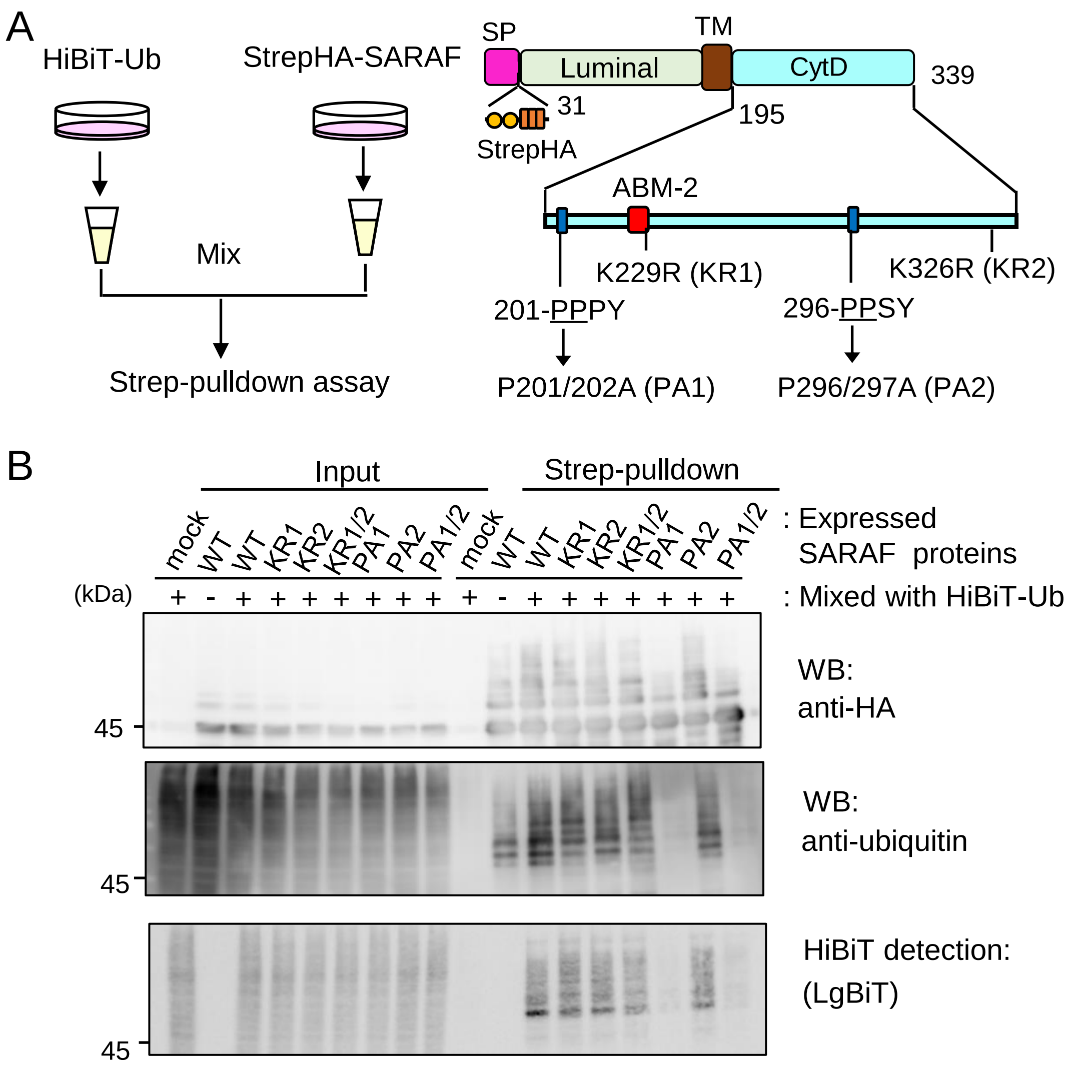

Supplementary Figure S4. Detection of post cell-lysis ubiquitination of full-length StrepHA-SARAF WT and mutants. (**A**) Flowchart of experiments and schematic diagram of full-length StrepHA-SARAF. The StrepHA tag was inserted between the C-terminus of the SARAF signal peptide (SP) sequence (1-30 a.a.) and the N-terminus of the mature SARAF (31-339 a.a.). Amino acid substitutions were introduced at two PPXY motifs (Pro-to-Ala mutations) and two potential ubiquitination sites (Lys-to-Arg mutations) in the SARAF CytD. (**B**) Cleared cell lysates of HEK293 cells expressing StrepHA-SARAF (WT, mutants or mock transfectant) were mixed with those expressing HiBiT-Ub or untransfected cells. The mixture (*Input*) and Strep-pulldown products were resolved by SDS-PAGE and analyzed by WB with anti-HA and anti-ubiquitin mAbs, respectively, as indicated or analyzed by probing with LgBiT for detection of HiBiT-Ub.

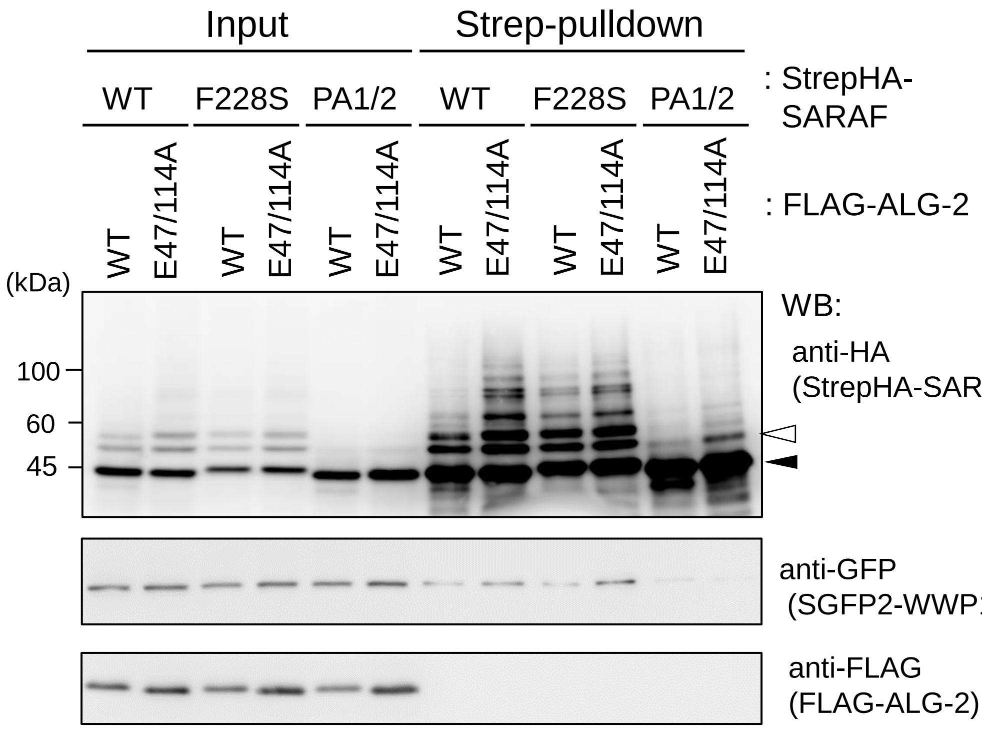

Supplementary Figure S5. Suppression of WWP1-dependent in-cell ubiquitination of SARAF by overexpressing ALG-2. HEK293 ALG-2KO cells were co-transfected with StrepHA-SARAF, SGFP2-WWP1 and WT or Ca^{2+}-binding-defective E47/114A mutant of FLAG-ALG-2. The Strep-pulldown assay was performed by using cell lysates prepared in a buffer containing ubiquitin E3 ligase inhibitors (5 mM EDTA and 10 mM NEM). The cleared cell lysates and pulldown products were analyzed by WB with mAbs against HA, GFP and FLAG as indicated. FLAG-ALG-2 was not detected in the pulldown products due to the presence of 5 mM EDTA in the lysis buffer.

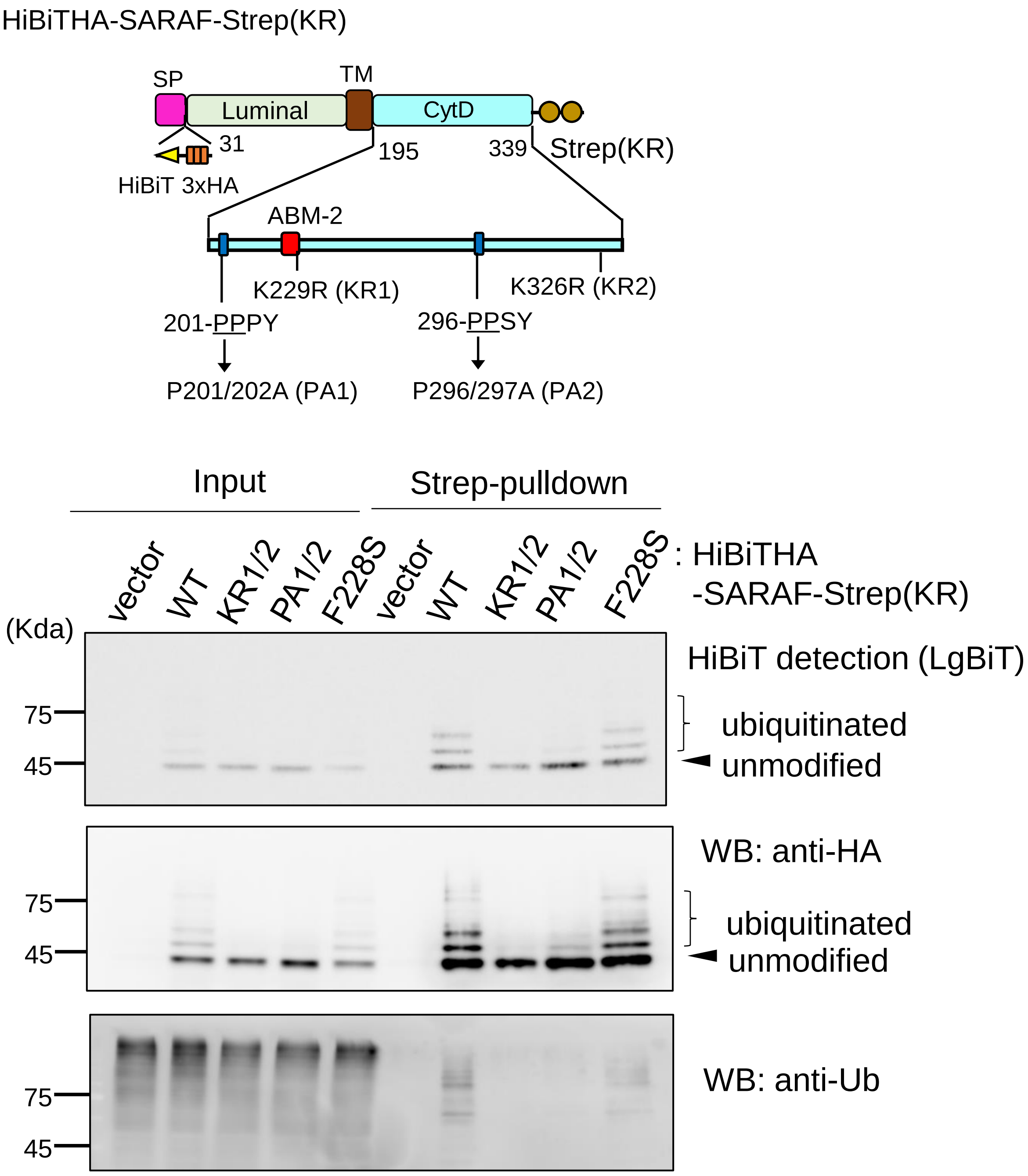

Supplementary Figure S6. LgBiT blotting (HiBiT detection) of full-length SARAF proteins tagged with HiBiTHA and Strep(KR). HEK293 ALG-2KO cells were co-transfected with expression plasmids for WT or mutants of HiBiTHA-SARAF-Strep(KR) and for SGFP2-WWP1. One day after transfection, cells were lysed with lysis buffer HK containing 1% Nonidet P-40 and protease inhibitors as well as ubiquitin E3 ligase inhibitors (2 mM EDTA and 10 mM NEM). The cleared cell lysates and Strep-pulldown products were subjected to blot analysis with LgBiT, anti-HA mAb and anti-ubiquitin mAb as indicated.
