# Supplementary material for: The Penta-EF-Hand ALG-2 Protein Interacts with the Cytosolic Domain of the SOCE Regulator SARAF and Interferes with Ubiquitination
Source: Int J Mol Sci. 2020 Aug 31;21(17):6315. doi: 10.3390/ijms21176315 (PMC7504102; doi:10.3390/ijms21176315)
Supplement: Supplementary file 1 [file ijms-21-06315-s001.zip › SupplTables.pdf]

Supplementary Table S1. Summary of ALG-2-interacting protein candidates analyzed in this article

Results of *in vitro* binding assays shown in Figure 1 are summarized. Fragments of proteins containing ABM-2 sequences were expressed and analyzed for binding with Nluc-ALG-2 by Far-western (FW) and pulldown assays.

Group	Protein name	UniProt ID	Expressed region	ABM-2		Intrinsically disordered [c]	Interaction [d]	
				Sequence [a]	PRR [b]		FW	Pulldown
A	Sec31A	O94979	790 - 1095	838-**PPPPGFI**	y	Y	++	+++
	SARAF	Q96BY9	195 - 339	223-**PPPPGF**K		Y	+++	++
	SHISA4	Q96DD7	122 - 197	153-**PP**Q**PGFI**	y	Y	+	+
	cTAGE5	O15320	502 - 804	764-**PP**R**PGF**F	y	Y	++	+
B	FNDC3A	Q9Y2H6	59 - 250	117-**PP**L**PGFI**	y		+	+/-
	DIAPH3	Q9NSV4	550 - 636	613-**PPP**L**GF**L	y		+/-	-
C	SBF1	O95248	1119 - 1319	1313-**PP**D**PGF**L			-	-
	PIK3R4	Q99570	572 - 1034	974-**PPPPG**WR			-	-
	DMRTC2	Q8IXT2	150 - 271	206-**PP**F**PGF**D	y		-	-
	NR3C2	P08235	1 - 602	475-**PP**V**PGF**D			-	-
	CHRNA1	P02708	342 - 453	402-**PPP**M**GF**H			-	-
	STARD5	Q9NSY2	1 - 213	143-**PP**K**PGF**V			-	-
	CCER1	Q8TC90	1 - 406	94-**PPPPGF**W			-	-

[a] Residues identical with the ABM-2 sequence of Sec31A are shown in bold face.

[b] The letter y indicates that the ABM-2 sequence is contained in a Pro-rich region (PRR) annotated in the

[c] The letter Y indicates that the entire ABM-2 (7 a.a.) and its surounding sequences on both sides (21 a.a.) are predicted to be located in an intrinsically disordered region (See Supplementary Figure S1).

[d] Relative binding abilities compared to Sec31A are scored and idicated by +++ (very strong), ++ (strong), + (moderate) +/- (weak), and - (negative) in the binding assays by Far Western (FW) and pulldown followed by Nluc-ALG-2 binding.

Supplementary Table S2.

Protein name	UniProt I	Plasmid source	Nucleotide Sequence Acc. No.	clone ID	Expressed region	SGFP2-fusion (vector)	Full or partial	Forward primer	Reverse primer	Cloning site
CCER1	Q8TC90	RIKEN[1)]	BC024183	IRAK033D24	1-406	SGFP2-C1	FL	ctcaagcttcgaattccatgactcagaccctcgaca	ccgcggtaccgtcgacctaacagttaaagtcctgtgc	EcoRI/SalI
cTAGE5	O15320	RIKEN	BC064355	IRAK134N08	502-804	SGFP2-C1	P	ctcaagcttcgaattctccttatgcactcgatgttc	ccgcggtaccgtcgactgtcaggtttcttgctgtgg	EcoRI/SalI
CHRNA1	P02708	NBRC	AK299445	FLJ58615	342-453	SGFP2-C1	P	ctcaagcttcgaattccaacacacaccaccgctca	ccgcggtaccgtcgacctagtccatcaccattgcaacgt	EcoRI/SalI
DIAPH3	Q9NSV4	NBRC[2)]	AK092024	FLJ34705	550-636	SGFP2-C1	P	ctcaagcttcgaattctcagtttggtgccttgccag	ccgcggtaccgtcgacctatggtttcaacccaaatggc	EcoRI/SalI
DMRTC2	Q8IXT2	RIKEN	BC029202	IRAK051E15	150-271	SGFP2-C1	P	ctcaagcttcgaattcgaactcctgtgggcctctg	ccgcggtaccgtcgacctatggctgtagctgaagagg	EcoRI/SalI
FNDC3A	Q9Y2H6	NBRC	AK302415	FLJ32842	59-250	SGFP2-C1	P	ctcaagcttcgaattctggtcctgctcaggttccaa	ccgcggtaccgtcgacttatgtatcaacttgtgtaccac	EcoRI/SalI
NR3C2	P08235	NBRC	AK304318	FLJ34705	1-481	SGFP2-C1	P	ctcaagcttcgaattcgatggagaccaaaggctac	ccgcggtaccgtcgacacaccaatcatatctttgaaggtc	EcoRI/SalI
PIK3R4	Q99570	OBS[3)]	BC110318	MHS6278-202758656	572-1034	SGFP2-C1	P	ctcaagcttcgaattcgaaagccaacgatgttttgtt	ccgcggtaccgtcgacatagatcaggtagtggtggtc	EcoRI/SalI
SBF1	O95248	RIKEN	BC009268	IRAL022F18	1119-1596	SGFP2-C1	P	ctcaagcttcgaattcgagcagcctggtggaaagg	ccgcggtaccgtcgacctaagtgtagaagtcccacac	EcoRI/SalI
STARD5	Q9NSY2	RIKEN	BC004365	IRAL013P20	1-213	SGFP2-C1	FL	ctcaagcttcgaattcaatggacccagcactggca	ccgcggtaccgtcgactagcattactcatggaattg	EcoRI/SalI
SARAF	Q96BY9	OBS	BC015012	MHS6278-202756988	195-339	SGG-SGFP2-N1	P	ctcaagcttcgaattgtcatgagtgacgggcagta	gcctccgcttggatctcgtctcctggtaccaccata	EcoRI/BamHI
SARAF	Q96BY9	OBS	BC015012	MHS6278-202756988	1-339	SGG-SGFP2-N1	FL	ctcaagcttcgaattccatcttcatcgagcgccat	gcctccgcttggatctcgtctcctggtaccaccata	EcoRI/BamHI

[1)] Complete sequencing and characterization of 21,243 full-length human cDNAs.Ota T, Suzuki Y, Nishikawa T, Otsuki T, Sugiyama T, et al.Nat Genet. 2004 Jan;36(1):40-5. Epub 2003 Dec 21. PMID: 14702039

[2)] National Institute of Technolopgy and Evaluation (NITE) Biological Resource Center. https://www.nite.go.jp/nbrc/catalogue/NBRCDispSearchServlet?lang=en

[3)] Open Biosystems. https://horizondiscovery.com/en/about-us/about-open-biosystems
