# Supplementary material for: The Penta-EF-Hand ALG-2 Protein Interacts with the Cytosolic Domain of the SOCE Regulator SARAF and Interferes with Ubiquitination
Source: Int J Mol Sci. 2020 Aug 31;21(17):6315. doi: 10.3390/ijms21176315 (PMC7504102; doi:10.3390/ijms21176315)
Supplement: Supplementary file 1 [file ijms-21-06315-s001.zip › Suppl_Info.pdf]

# Supplementary Information

## Construction of plasmids

### pStrepHA-SGFP2

pStrepHA-SGFP2 (shortened name for pEXPR-IBA105-C/3xHA-SGG-SGFP2-N1) was constructed by multi-step manipulation of modifying mammalian epitope tag vectors (FLAG, Strep, HA, and SGFP2). First, a Strep vector pEXPR-IBA105-C was constructed by inserting an oligonucleotide block (5'-gaattcggggatccgatatctcgaggtacca-3'; 5'-agcttggtacctcgagatatcggatccccgaattcgc-3') at the SacII/HindIII site of pEXPR-IBA105 (IBA GmbH, Göttingen, Germany) to generate the multiple cloning sites with a different reading frame. Next, pCMV-3xHA-A was constructed by digesting pCMV-3xFLAG-A [Suppl. Ref 1] with NotI and BamHI to remove a 3xFLAG-encoding DNA fragment and inserting a pair of 2xHA-encoding oligonucleotides (5'-ggccgccaccatggtttacccatacgatgttcctgactatgctggctatccctatgacgtcccggactatgcag-3'; 5'-gatcctgcatagtccgggacgtcatagggatagccagcatagtcaggaacatcgtatgggtaaaccatggtggc-3') and then by further inserting a pair of 1xHA-encoding oligonucleotides (5'-gatcttatccatatgacgttccagattacgctccgg-3'; 5'-gatcccggagcgtaatctggaacgtcatatggataa-3') at the BamHI/BglII site. Human NFAT2 cDNA clones (FLJ76687, encoding 1-716 a.a. of UniProt ID O95644-2, NFATc1 Isoform A-alpha; FLJ61216, encoding 473-943 a.a. of UniProt ID O95644-1, NFATc1 Isoform C-alpha) were obtained from NITE Biological Resource Center (NBRC) and used as template DNAs to amplify an NFAT2 cDNA encoding 1-943 a.a. by the overlap PCR method to fuse 1-503 a.a. and 495-943 a.a. using two pairs of primers (Pair 1, forward, 5'-ttacgctccgggatccccaagcaccagctttccagt-3', reverse, 5'-tggacacggtcttccctgtgatgcggtg-3'; Pair 2, forward, 5'-caccgcatcacagggaagaccgtgtcca-3', reverse, 5'-cggtatcgataagcttctaggagtgggtgctcgtgc-3'; overlapped primers encoding 495-503 a.a. underlined) and inserted into the BamHI/HindIII site of pCMV-3xHA-A with an In-Fusion® HD Cloning Kit (Clontech/TakaraBio). The resultant plasmid pCMV-3xHA-A/NFAT2 (1-943) was used for construction of pStrepHA-NFAT2 by inserting a PCR-amplified DNA fragment (primers: 5'-ccgagaccgcgaattcgaccatggtttacccatacgat-3'; 5'-ctgcggccgcaagcttcgaggtcgacggtatcgata-3') into the EcoRI/HindIII site of the above described pEXPR-IBA105-C. The resultant pStrepHA-NFAT2(1-943) was digested with BamHI and HindIII to remove the NFAT2 cDNA fragment, followed by insertion of a cDNA fragment encoding SGFP2 using pSGG-SGFP2-N1 [Suppl. Ref 2] as a template and a pair of primers (forward, 5'-ttacgctccgggatctgagctcaagcttcgaattctgc-3'; reverse, 5'-

ctgcggccgcaagctacgctttacttgtacagctcgtc-3’) in which the HindIII site downstream of the SGFP2-encoding DNA segment was destructed.

**pSP-StrepHA-SARAF**

To construct pSP-StrepHA-SARAF, a synthetic DNA block encoding the SARAF signal peptide sequence (1-MAAACGPGAAGYCLLLGLHLFLLTAGPALG-30) was first inserted between the XbaI and NheI sites of pStrepHA-SGFP2 using the following two pairs of partially complementary oligonucleotides: SigPep-F1, 5’-ctagacccaccatggctgcagcatgcggacctggagcagcaggctactgcttgctcctgg-3’, SigPep-F2, 5’-gactgcatctgttcctgctgacagctggacctgcactgggtg-3’; SigPep-R1, 5’-ctgctgctccaggtccgcatgctgcagccatggtgggt-3’, SigPep-R2, 5’-ctagcacccagtgcaggtccagctgtcagcaggaacagatgcagtcccaggagcaagcagtagc-3’ (complementary sequence in the primers SigPep-F1 and SigPep-R2 underlined; SigPep-F1 and SigPep-R2 phosphorylated by the T4 kinase reaction *in vitro*). The resultant plasmid pSP-StrepHA-SGFP2 (shortened name for pEXPR-IBA105-C/SARAF(1-30) 3xHA-SGG-SGFP2) enabled secretion of StrepHA-SGFP2 into the culture medium from transfected HEK293T cells. For replacement of the SGFP2-encoding DNA segment of pSP-StrepHA-SGFP2 with the DNA fragment encoding SARAF 31-339 a.a., In-Fusion PCR was performed with a pair of primers (forward, 5’-atctgagctcaagctttggaacgaccctgacagaatg-3’; reverse, 5’-gcggtttaaacttaattatcgtctcctggtaccacc-3’, complementary sequence of re-created stop codon underlined) using pSGG-SGFP2-N1/SARAF as a template, and the amplified DNA was subcloned into the HindIII/AflII site of pSP-StrepHA-SGFP2, in which the HindIII site and the AflII site were located upstream and downstream of the SGFP2-encoding DNA segment. The mature protein encoded by pSP-StrepHA-SARAF is designated StrepHA-SARAF in this article.

**pSP-HiBiT-3xHA-SARAF**

HiBit-3xHA- and TwinStrep-tagging vector for single-pass transmembrane (TM) proteins (pCDNA3.1-SP-HiBiT-HA_TM_TwinStrepKR; sequences for signal peptide and TM from SARAF) was prepared by a gene synthesis service at GenScript (Piscataway, NJ, USA). A SARAF cDNA fragment without a stop codon was inserted between the BamHI site (located upstream of the TM region) and the EcoRV site (located between TM and TwinStrepKR) to construct an expression plasmid for SP-HiBiT-3xHA-SARAF-TwinStrep that was designed for assays combining Strep-pulldown and HiBiT detection.

A stop codon was re-introduced to express C-terminally untagged SARAF (SP-HiBiT-HA-SARAF). The mature protein name encoded by pSP-HiBiT-3xHA-SARAF is designated HiBiTHA-SARAF in this article.

**pCX4-hygro**

A retrovirus expression vector containing the hygromycin resistance (*hygro*) gene was constructed by replacing the blasticidin resistance (*bsr*) gene of pCX4-bsr [Suppl. Ref 3] with the *hygro* gene in pNL(NlucP/CRE/Hygro) (Promega) as briefly described below. pCX4-bsr was digested with NcoI and SalI to remove the *bsr* gene, and the remaining large vector fragment was isolated and ligated with the *hygro* gene by the Infusion-PCR method using a pair of specific primers (forward, 5’-aaaacacgataataccatgaagaagcccgaactcac-3’; reverse, 5’-ttttattttatcgtgtcgacgtttaaactcgacctacctccttagcg-3’, the *hygro*-encoding sequence and the sequence complementary to the *hygro*-encoding DNA underlined and double-underlined, respectively).

**Preparation of rabbit polyclonal antibodies**

**anti-SARAF pAb**

A cDNA fragment encoding SARAF CytD (SARAF195-339 a.a.) was inserted into the EcoRI/SalI site of the bacterial expression vector pGEX-4T-1 (GE Healthcare) by the In-Fusion PCR method using a pair of primers (forward, 5'-tggatccccggaattcagtgacgggcagtattctcc-3'; reverse, 5'-ggccgctcgagtcgactttatcgtctcctggtacca-3'). Using the resultant plasmid pGST-SARAF-Ct as a template, an internal sequence (corresponding to 225-278 a.a.) was deleted by the QuikChange site directed mutagenesis method using a pair of primers (forward, 5'-accaactcagcaggacctcctagcaatagagcggcaacaccc-3'; reverse, 5'-gggtgttgccgctctattgctaggaggtcctgctgagttggt-3') to improve solubility of the expressed recombinant protein GST-SARAF-Ct-4 (expressing 195-224/278-339 of SARAF) in *Escherichia coli*. To further increase the solubility, a DNA fragment encoding GST-SARAF-Ct-4 was transferred to the EcoRI/SalI site of the cold shock vector pColdII (TakaraBio) by the In-Fusion PCR method using a pair of primers (forward, 5'-cgagggatccgaattcatgtcccctatactagg-3'; reverse, 5'-tagactgcaggtcgactttatcgtctcctg-3') and pGST-SARAF-Ct-4 as a template. For expression of maltose-binding protein (MBP)-fused SARAF-Ct-4, pGST-SARAF-Ct-4 was digested with EcoRI and SalI, and a DNA fragment encoding SARAF-Ct-4 was subcloned into the EcoRI/SalI site of pMal-c2

(NEB, Ipswich, MA) to construct pMAL-c2/SARAF-Ct-4.

*Escherichia coli* BL21 cells were transformed with pColdII/GST-SARAF-Ct-4 and pre-cultured at 37 $^{o}$C overnight in LB broth containing 50 μg/mL of ampicillin. Fresh culture was diluted 1:100 with 300 ml of ZYG broth containing 0.4% glucose and ampicillin, and incubated with aeration at 37 $^{o}$C. When absorbance of the culture at 600 nm reached 0.3-0.5, culturing temperature was shifted down to 15 $^{o}$C. After 30 min, IPTG was added to 0.5 mM, and incubation was continued overnight. Harvested cells were lysed by sonication in lysis buffer (20 mM Tris-HCl, pH 8.0, 150 mM NaCl, 5 mM EDTA, 2 mM 2-mercaptoethanol, 1 mM PMSF, 5 mM benzamidine) containing 1% Triton-X100 and processed for purification with glutathione Sepharose beads (GE Healthcare) essentially according to the provided manufacturer's instructions. After the glutathione-eluted fractions were dialyzed against phosphate buffered saline, the purified GST-SARAF-Ct-4 protein was used for immunization of rabbits.

*Escherichia coli* BL21 cells transformed with pMAL-c2/SARAF-Ct-4 were cultured at 30 $^{o}$C and induced for expression with 0.3 mM IPTG for 3 h. Harvested cells were lysed in lysis buffer (20 mM Tris-HCl, pH7.5, 200 mM NaCl, 1 mM EDTA, 1 mM dithiothreitol, 1 mM PMSF) containing lysozyme for 20 min on ice followed by sonication. The MBP-SARAF-Ct-4 protein was affinity purified with an amylose resin column. Maltose-eluted fractions were dialyzed against water and used for immobilization to a HiTrap NHS-activated HP Column (1-mL size, GE Healthcare) according to the manufacturer's instructions. The column was pre-washed with 10 mL of 0.1 M Glycine-HCl, pH 3.0, and renatured with 10 mL of TBS before use. The rabbit antiserum against GST-SARAF-Ct-4 was diluted with TBS and absorbed to the column. After the fractions eluted with 0.1 M Glycine-HCl, pH 3.0, was neutralized with 1 M Tris-HCl, pH 9.0, and dialyzed against PBS, antibodies cross-reacting with GST and MBP were removed by successive incubation with GST-immobilized Sepharose beads and MBP-immobilized amylose resin.

**anti-GFP pAb**

A cDNA fragment encoding monomeric EGFP (mEGFP; A206K mutant of EGFP, initiation Met not counted for residue Nos.; see Suppl. Refs 4 and 5.) was obtained by PCR-amplification with a pair of primers (forward, 5'-ggttccgcgtggatccatggtgagcaagggcga-3', EGFP-encoding sequence including ATG underlined; reverse, 5'-ggccgctcgagtcgactagtacagctcgtccatgc-3', the nucleotide sequence complementary to the C-terminal EGFP-encoding sequence followed by TAG double-underlined) and using pmEGFP-C1 as a template, and it was subcloned into the

BamHI/SalI site of pGEX-4T-3. The resultant plasmid pGEX-4T-3/mEGFP was used for bacterial expression of GST-GFP in *Escherichia coli* BL21. The BamHI/SalI fragment of pGEX-4T-3/mEGFP was further subcloned into the BamHI/SalI site of pMAL-c2 to construct pMAL-c2/mEGFP, which was used for bacterial expression of MBP-GFP. Bacterial expression and purification of the GST-GFP and MBP-GFP proteins were performed by conventional methods as described elsewhere. While GST-GFP was used for immunization of rabbits, MBP-GFP was immobilized to a HiTrap NHS-activated HP Column (1-mL size) and used for affinity purification of anti-GFP pAb as similarly described for preparation of anti-SARAF pAb.

**Supplementary References**

1. Katoh K, Shibata H, Suzuki H, Nara A, Ishidoh K, Kominami E, Yoshimori T, Maki M. (2003) The ALG-2-interacting protein Alix associates with CHMP4b, a human homologue of yeast Snf7 that is involved in multivesicular body sorting. J Biol Chem. 278(40):39104-13. [PubMed ID, 12860994]

2. Shibata H, Suzuki H, Yoshida H, Maki M. (2007) ALG-2 directly binds Sec31A and localizes at endoplasmic reticulum exit sites in a Ca2+-dependent manner. Biochem Biophys Res Commun. 353(3):756-63. [PubMed ID, 17196169]

3. Akagi T, Sasai K, Hanafusa H. (2003) Refractory nature of normal human diploid fibroblasts with respect to oncogene-mediated transformation. Proc Natl Acad Sci U S A. 100(23):13567-72. [PubMed ID, 14597713]

4. Zacharias DA, Violin JD, Newton AC, Tsien RY. (2002) Partitioning of lipid-modified monomeric GFPs into membrane microdomains of live cells. Science. 296(5569):913-6. [PubMed ID, 11988576]

5. Yang F, Moss LG, Phillips GN Jr. (1996) The molecular structure of green fluorescent protein. Nat Biotechnol. 14(10):1246-51. [PubMed ID, 9631087]
